# A case report: subacute combined degeneration of the spinal cord and pernicious anemia caused by autoimmune gastritis

**DOI:** 10.1097/MD.0000000000029226

**Published:** 2022-06-30

**Authors:** Zhihuan Sun, Xiaofei Yu

**Affiliations:** From the Department of Neurology, Shuguang Hospital Affiliated to Shanghai University of Traditional Chinese Medicine, Pudong New District, Shanghai, China.

**Keywords:** adenosylcobalamin, autoimmune gastritis, methylcobalamin, subacute combined degeneration of the spinal cord, vitamin B12 deficiency

## Abstract

Autoimmune gastritis causing both subacute combined degeneration of the spinal cord and pernicious anemia is rare in clinical practice. Here, we report a case of subacute combined degeneration of the spinal cord and pernicious anemia resulting from vitamin B12 deficiency due to autoimmune gastritis.

**Patient concerns:**

A 66-year-old woman presented with a 2-month history of numbness in her extremities.

**Diagnoses:**

The diagnoses were (1) autoimmune gastritis (2) subacute combined degeneration of the spinal cord (3) pernicious anemia (4) hypergastrinemia (5) chronic lymphocytic thyroiditis.

**Interventions:**

The patient received intramuscular methylcobalamin treatment for 5 days, followed by oral methylcobalamin daily.

Outcomes: Symptoms improved, and anemia recovered in the second month after discharge. She discontinued her medication afterward, and the neurological symptoms recurred.

**Conclusions:**

Autoimmune gastritis can lead to several diseases if not intervened in the early course. Neuropathy and hematopathy recur with treatment discontinuity. Methylcobalamin and adenosylcobalamin are unlikely to be more effective than vitamin B12.

## 1. Introduction

Subacute combined degeneration (SCD) of the spinal cord is induced by vitamin B12 deficiency and is characterized by demyelination of the posterior columns of the cervical and/or dorsal cord. Furthermore, pernicious anemia (PA) is associated with vitamin B12 deficiency. There are numerous causes of vitamin B12 deficiency including low dietary intake, malabsorption (autoimmune gastritis, ileal disease, etc), gastric and intestinal surgery, inherited disorders, and medications.^[1]^ In this case, autoimmune gastritis (AIG) was the cause of vitamin B12 deficiency and caused both neuropathy and hematopathy, which is rare in clinical practice.

Herein, we report a case of a 66-year-old woman who experienced subacute combined degeneration of the spinal cord and pernicious anemia resulting from vitamin B12 deficiency due to autoimmune gastritis.

## 2. Case Report

A 66-year-old woman was admitted to our neurology department with concern of numbness in her extremities. She presented with lateral hallux numbness 2 months prior, which progressively extended to the lateral vola and other toes in a stocking distribution. Without seeking medical treatment, she had experienced numbness in the lateral fingers in a glove distribution accompanied by weakness and coldness in all limbs 1 month earlier. One week before administration, she visited a neurology outpatient clinic in our hospital, where she was prescribed 500 μg/day of vitamin B12 intramuscularly for 2 days and vitamin B6 10 mg/day folic acid 5 mg/day orally for a week. Symptoms were not relieved after a week. Neurological examinations revealed decreased superficial sensory perception in the distal extremities and a sense of position in both lower extremities. In addition, the patient presented with unsteadiness in the heel-to-toe walking test. The deep tendon reflexes were normal, along with the results of the other examinations. She had no significant medical history, unbalanced diet, alcohol consumption, or drug misuse.

**Figure 1.**
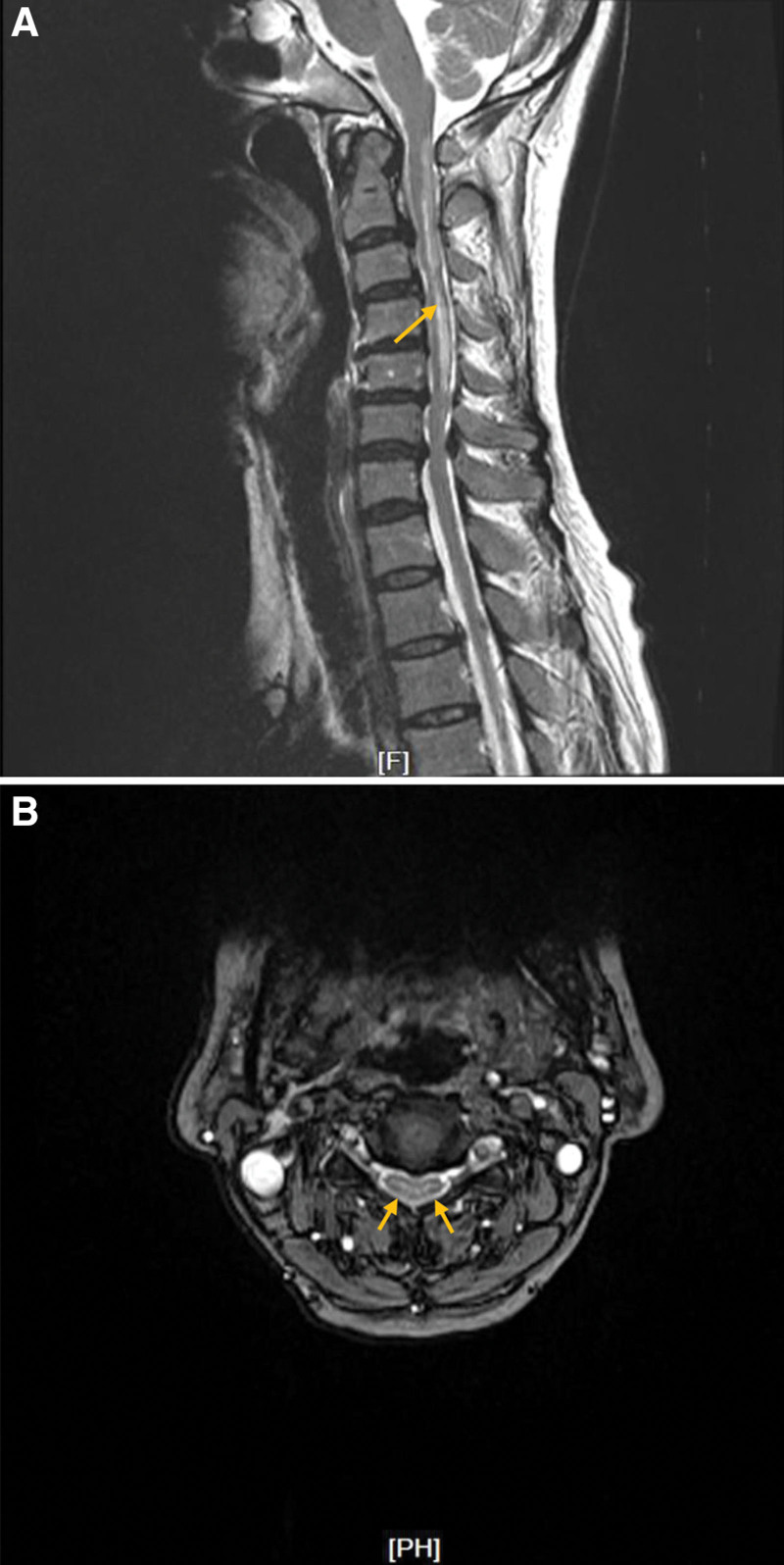
The cervical spine magnetic resonance imaging. A: A T2-weighted image showing the high-intensity area in the posterior column at C2 through C5 (yellow arrow). B: An axial T2-weighted image of the cervical spinal cord showing bilaterally symmetrical high-intensity area within the posterior column (yellow arrows).

**Figure 2.**
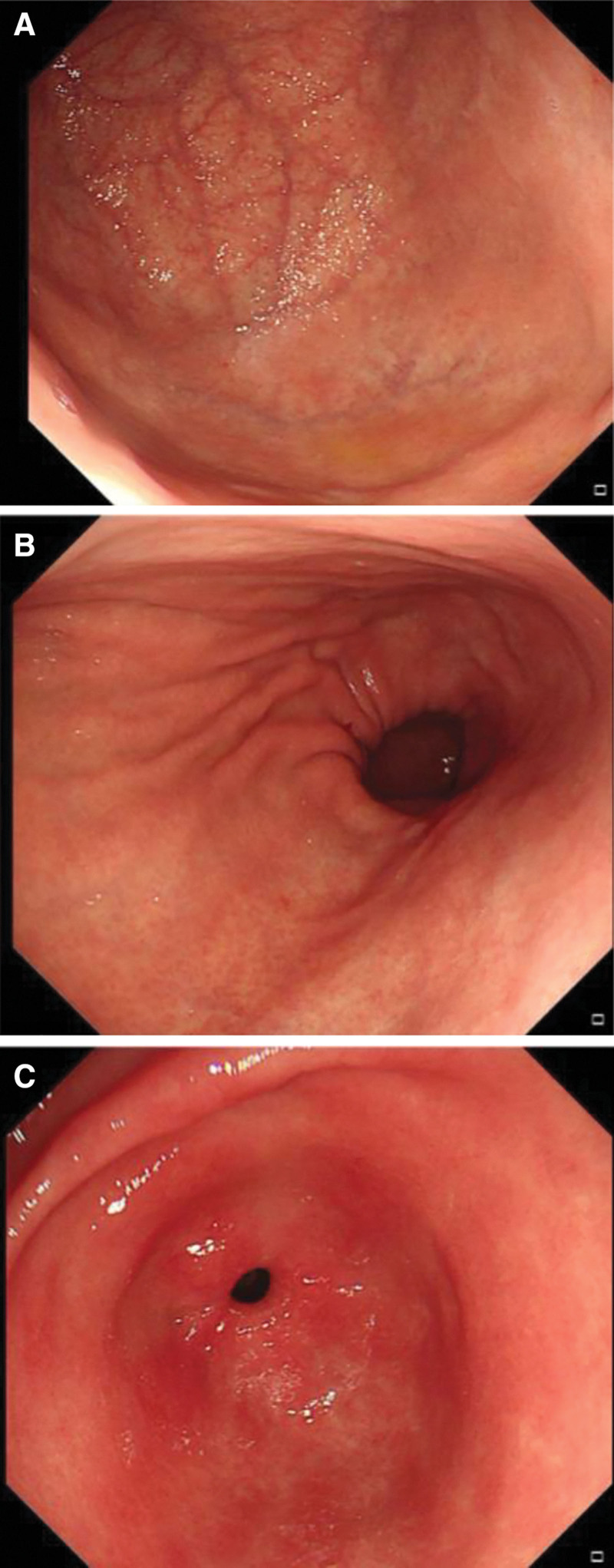
Gastroduodenoscopy findings. A and B: Atrophic gastritis in the corpus and fundus of the stomach. C: Mild hyperemic and exudative gastritis in the antrum. There was no evidence of gastric cancer or carcinoid.

The laboratory diagnostics showed reduced erythrocytes (2.45 × 10^12^/L, normal range 3.68–5.13 × 10^12^/L), hemoglobin (94 g/L, normal range 113–151g/L) and leukocyte (2.54 × 10^9^/L, normal range 3.69–9.16 × 10^9^/L); increased mean corpuscular volume (117.1 fL, normal range 82.6–99.1 fL) and mean corpuscular hemoglobin (38.4 pg, normal range 26.9–33.3 pg); highly increased level of serum vitamin B12 (>1500 pg/mL, normal range 180–914 pg/mL) and folic acid (>22.8 ng/mL, normal range 3.1–19.9 ng/mL); decreased pepsinogen-I level (11.2 ng/mL, normal range 33–110 ng/mL) and pepsinogen-I/pepsinogen-II ratio (1.7, normal range >3.0); highly elevated gastrin-17 level (125.6 pmol/L, normal range 1.7–7.6 pmol/L); normal homocysteine level (7.1 µmol/L, normal range 0–20 µmol/L); positive antiintrinsic factor antibodies (IFA); negative 13C-urea breath test and anti-*Helicobacter pylori* antibody. Furthermore, her antithyroid peroxidase antibody level was elevated, although the thyroid-stimulating hormone, free T4, and T3 levels were within the reference range. There were high-intensity areas in the posterior column of the cervical cord through C2 to C5 on T2-weighted magnetic resonance imaging (MRI) of the cervical spine. (Fig. 1) No significant abnormality was found on brain MRI. Motor and sensory nerve conduction velocities were also unremarkable. Gastroduodenoscopy revealed atrophic gastritis in the corpus and fundus, whereas mild hyperemic and exudative gastritis were observed in the antrum. (Fig. 2) Examination of the biopsy specimen showed chronic inflammation of the superficial mucosa from the antrum of the stomach, chronic atrophic gastritis and mild intestinal metaplasia from the corpus, and chronic atrophic gastritis from the fundus.

The diagnoses were (1) autoimmune gastritis (2) subacute combined degeneration of the spinal cord (3) pernicious anemia (4) hypergastrinemia (5) chronic lymphocytic thyroiditis. AIG was confirmed by gastroduodenoscopy which was performed for strong relationships among AIG, vitamin B12 deficiency, and positive IFA.^[2]^ Both gastroscopic and pathological results revealed chronic atrophic gastritis in the corpus and fundus with antral sparing, a typical histologic manifestation of AIG.^[3]^ H. pylori infection was ruled out. The diagnosis of SCD was supported by cervical spine MRI and clinical improvement after 2-month of supplementation despite the elevated serum vitamin B12 level. Blood samples were collected after 1 week of replenishing treatment in the outpatient clinic and no more records before that.

The patient was treated with 1000 μg/day of methylcobalamin (MeCbl) intramuscularly for 5 days during hospitalization and was prescribed 1500 μg/day orally every day after discharge. During the 3-month follow-up visit, we were told that all her symptoms, including numbness, weakness, and ataxia, improved in the second month after she was discharged. However, she discontinued her medication afterward and relapsed. Even so, routine blood examination showed recovery from anemia at that visit. Owing to her reluctance for additional injection treatment, the doctor prescribed intramuscular adenosylcobalamin (AdoCbl) 1500 μg once on that day and oral AdoCbl 1500μg/day every day with lifelong supplementation caution.

## 3. Discussion

The diagnoses in this case shows a correlation. Autoimmune gastritis is an inflammatory reaction in the gastric corpus and fundus due to CD4+ T cells attacking the H+/K+-ATPase proton pump on the luminal side of parietal cells, which results in B cells producing IFA and antiparietal cell antibodies (PCA), in turn contributing to atrophic progression in the gastric corpus and fundus.^[4]^ PCA decreases the secretion of stomach acids from oxyntic glands. Achlorhydria stimulates the gastrin-secreting cells resulting in chronic hypergastrinemia. In hypergastrinemia conditions, the life of endocrine-like cells is prolonged, which raises the possibility of neuroendocrine tumors.^[3]^ Meanwhile, elevated IFA renders vitamin B12 uptake in the gastrointestinal tract and gives rise to its insufficiency.

Vitamin B12 is a cofactor of methionine synthase and methylmalonyl-CoA mutase.^[1]^ Its deficiency contributes to reduced methionine synthase activity, which in turn generates myelin synthesis disorder as SCD, in which the thickest myelin sheaths (such as posterior and pyramidal lateral columns) are preferentially affected.^[5]^ Vitamin B12 deficiency also results in macrocytic anemia (PA in this case) which is associated with decreased thymine synthesis. The underlying mechanisms involve impaired DNA synthesis and transcription. The maturation of the cytoplasm and nuclei is asynchronous, leading to macrocytosis and immature nuclei in the peripheral blood.^[1]^

Autoimmune thyroid diseases such as Hashimoto thyroiditis and, to a lesser extent, Grave disease, are the most associated autoimmune comorbidities with AIG.^[6–8]^ In our case, the patient was diagnosed with Hashimoto thyroiditis. Therefore, screening for autoimmune thyroid disease is recommended in patients diagnosed with AIG.^[2]^

The resolution of SCD and PA caused by AIG is adequate supplementation. Both parenteral vitamin B12 treatment (8–10 loading injections of 1000μg each, followed by monthly 1000-μg injections) and high-dose oral vitamin B12 treatment (1000–2000 μg daily) are comparable effective therapies.^[1,9,10]^ What’s more, treatment is lifelong in those who suffer neurological and hematological manifestations from AIG. It has been proven that neurological symptoms recur within 6 months and macrocytic anemia recurs in several years with discontinuity of vitamin B12 supplementation after clinical recovery.^[1]^

The peculiarity in this case is that different doctors prescribed diverse forms of cobalamin (Cbl). MeCbl and AdoCbl are active forms of Cbl and are known to perform better than vitamin B12 in terms of efficacy. Is it true or not?

There are 4 forms of Cbl: cyanocobalamin (CNCbl), hydroxylcobalamin (HOCbl), methylcobalamin, and adenosylcobalamin. CNbl, also known as vitamin B12, is water-soluble. MeCbl and AdoCbl are functional coenzymes of methionine synthase and methylmalonyl-CoA mutase, respectively. HOCbl is a physiologically relevant intermediate form.

All forms of enteral Cbl follow identical steps when trafficking into mammalian cells: crossing the intestinal barrier into the blood, cellular internalization, lysosomal release, dealkylation, decyanation or reduction, forming the coenzymes, and then attaching to the corresponding enzymes. Parenteral Cbl also follows the same cellular internalization and subsequent steps.^[11]^

After internalization and lysosomal release, Cbl binds to the cytosolic chaperone MMACHC, which is responsible for converting all forms of Cbl into the common intermediate [Co2+] Cbl. The bound enables the decyanation of [CN-Co3+]Cbl to [Co2+]Cbl, dealkylation of MeCbl and AdoCbl to [Co2+/1+]Cbl, and reduction of HOCbl.^[12–14]^ Reduced [Co2+]Cbl is then delivered to either the cytoplasm for the formation of MeCbl or mitochondria for the formation of AdoCbl.^[15,16]^

MMACHC showed broad specificity for all Cbl forms in supplying the [Co2+]Cbl intermediate. This suggests that MeCbl and AdoCbl follow the same intracellular processing route as CNCbl.^[11]^ Moreover, there is no clinical evidence for the claimed strengths of MeCbl and AdoCbl. Thus, we conclude that supplementing MeCbl or AdoCbl is not advantageous compared to CNCbl.

In short, AIG causes and is correlated to many diseases if not intervened in early course. In vitamin B12 deficiency caused by AIG, enteral and parenteral treatments are proportionally effective and lifelong medication must be implemented. Until further evidence is available, MeCbl or AdoCbl is unlikely to be superior to vitamin B12.

### Author contributions

Zhihuan Sun performed the anlalysis of the reported case and wrote the manuscript. Xiaofei Yu contributed to the conception of the reported case and manuscript preparation.

## References

[1] Stabler SP. Clinical practice. Vitamin B12 deficiency. N Engl J Med. 2013;368:149–60. 23301732 10.1056/NEJMcp1113996

[2] Shah SC Piazuelo MB Kuipers EJ. AGA plinical practice update on the diagnosis and management of atrophic gastritis: expert review. Gastroenterology. 2021;161:1325–1332.e7 e7. 34454714 10.1053/j.gastro.2021.06.078 PMC8740554

[3] Neumann WL Coss E Rugge M. Autoimmune atrophic gastritis—pathogenesis, pathology and management. Nat Rev Gastroenterol Hepatol. 2013;10:529–41. 23774773 10.1038/nrgastro.2013.101

[4] Lenti MV Rugge M Lahner E. Autoimmune gastritis. Nat Rev Dis Primers. 2020;6:56. 32647173 10.1038/s41572-020-0187-8

[5] Scalabrino G. Cobalamin (vitamin B(12)) in subacute combined degeneration and beyond: traditional interpretations and novel theories. Exp Neurol. 2005;192:463–79. 15755562 10.1016/j.expneurol.2004.12.020

[6] Kalkan C Soykan I. Polyautoimmunity in autoimmune gastritis. Eur J Intern Med. 2016;31:79–83. 27085391 10.1016/j.ejim.2016.03.025

[7] Lahner E Centanni M Agnello G. Occurrence and risk factors for autoimmune thyroid disease in patients with atrophic body gastritis. Am J Med. 2008;121:136–41. 18261502 10.1016/j.amjmed.2007.09.025

[8] Miceli E Lenti MV Padula D. Common features of patients with autoimmune atrophic gastritis. Clin Gastroenterol Hepatol. 2012;10:812–4. 22387252 10.1016/j.cgh.2012.02.018

[9] Bolaman Z Kadikoylu G Yukselen V. Oral versus intramuscular cobalamin treatment in megaloblastic anemia: a single-center, prospective, randomized, open-label study. Clin Ther. 2003;25:3124–34. 14749150 10.1016/s0149-2918(03)90096-8

[10] Castelli MC Friedman K Sherry J. Comparing the efficacy and tolerability of a new daily oral vitamin B12 formulation and intermittent intramuscular vitamin B12 in normalizing low cobalamin levels: a randomized, open-label, parallel-group study. Clin Ther, 2011;33:358–371.e2 e2. 21600388 10.1016/j.clinthera.2011.03.003

[11] Obeid R Fedosov SN Nexo E. Cobalamin coenzyme forms are not likely to be superior to cyano- and hydroxyl-cobalamin in prevention or treatment of cobalamin deficiency. Mol Nutr Food Res. 2015;59:1364–72. 25820384 10.1002/mnfr.201500019 PMC4692085

[12] Gherasim C Lofgren M Banerjee R. Navigating the B(12) road: assimilation, delivery, and disorders of cobalamin. J Biol Chem. 2013;288:13186–93. 23539619 10.1074/jbc.R113.458810 PMC3650358

[13] Koutmos M Gherasim C Smith JL. Structural basis of multifunctionality in a vitamin B12-processing enzyme. J Biol Chem. 2011;286:29780–7. 21697092 10.1074/jbc.M111.261370 PMC3191019

[14] Li Z Gherasim C Lesniak NA. Glutathione-dependent one-electron transfer reactions catalyzed by a B(1)(2) trafficking protein. J Biol Chem. 2014;289:16487–97. 24742678 10.1074/jbc.M114.567339 PMC4047415

[15] Coelho D Kim JC Miousse IR. Mutations in ABCD4 cause a new inborn error of vitamin B12 metabolism. Nat Genet. 2012;44:1152–5. 22922874 10.1038/ng.2386

[16] Mah W Deme JC Watkins D. Subcellular location of MMACHC and MMADHC, two human proteins central to intracellular vitamin B(12) metabolism. Mol Genet Metab. 2013;108:112–8. 23270877 10.1016/j.ymgme.2012.11.284

